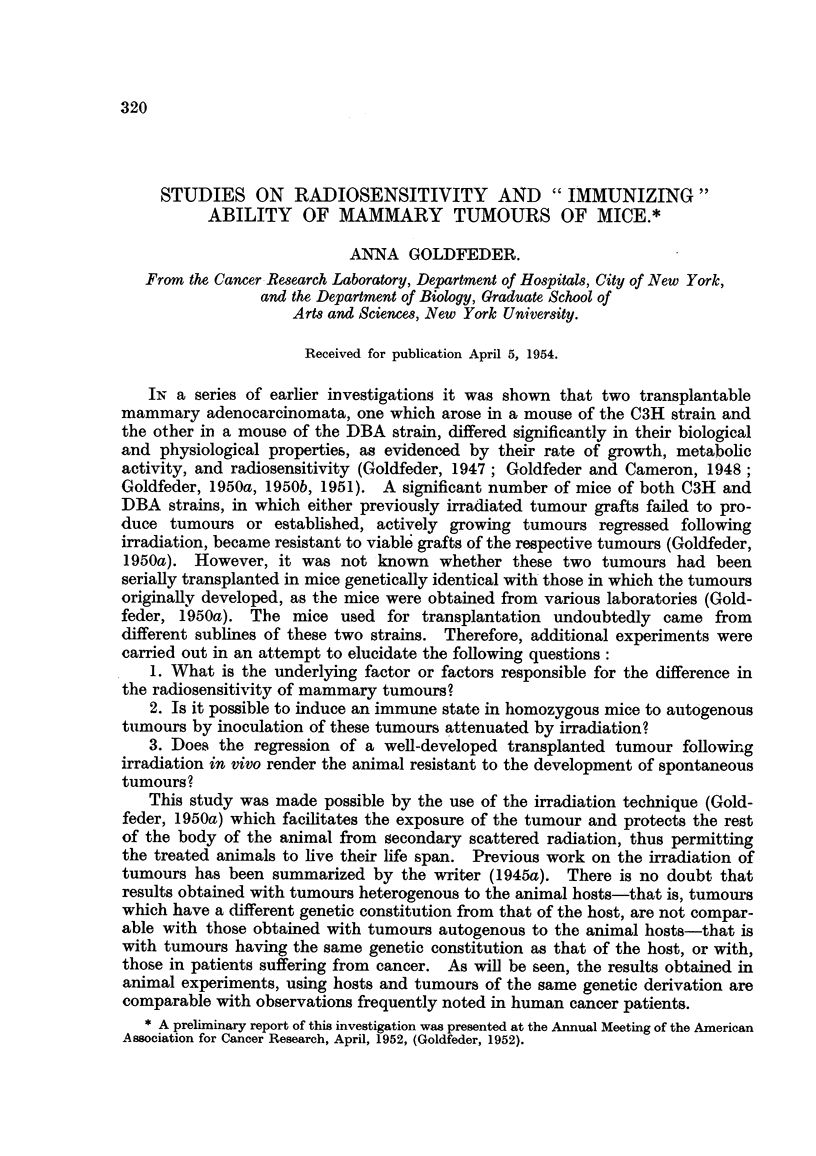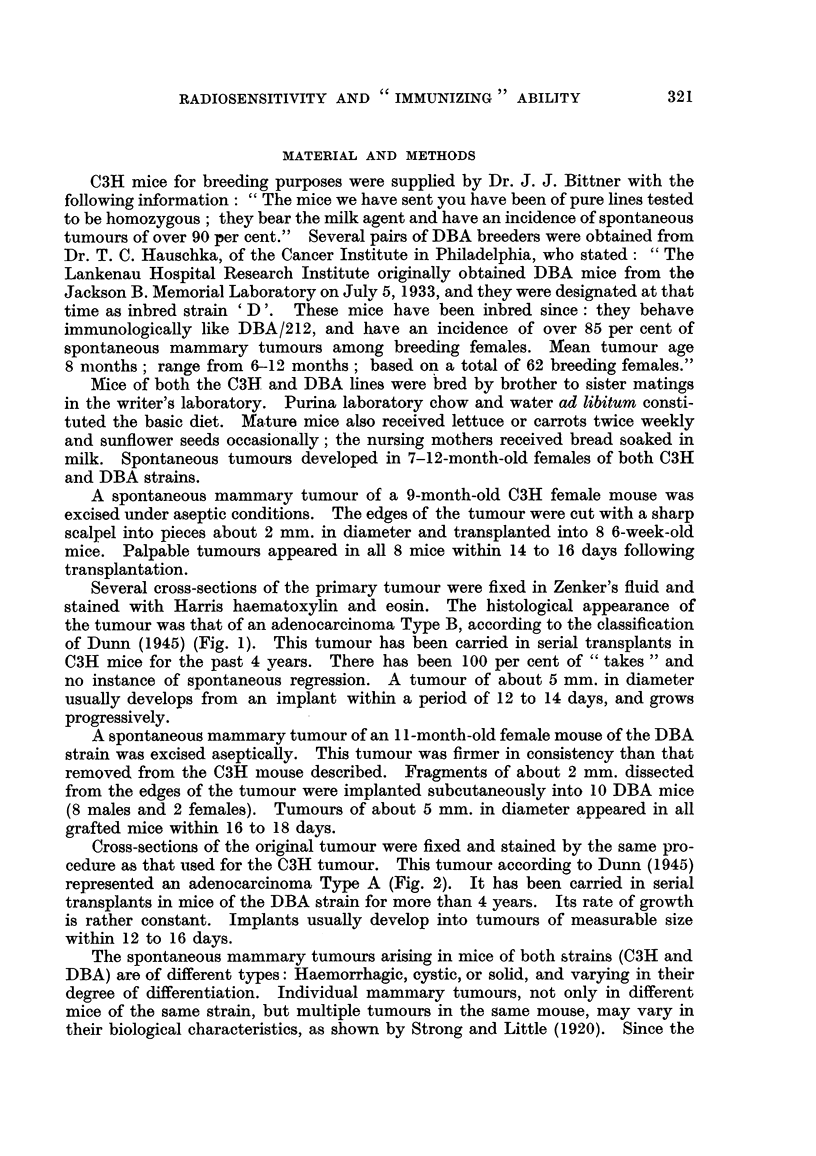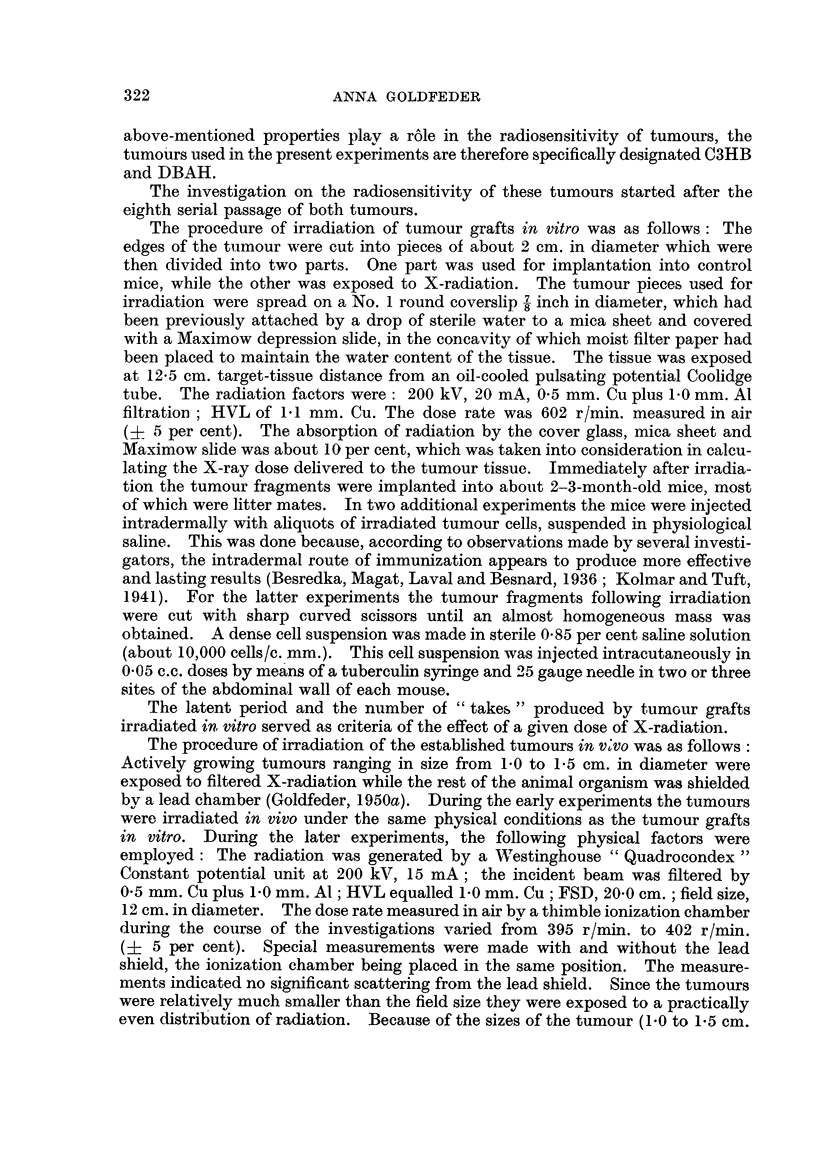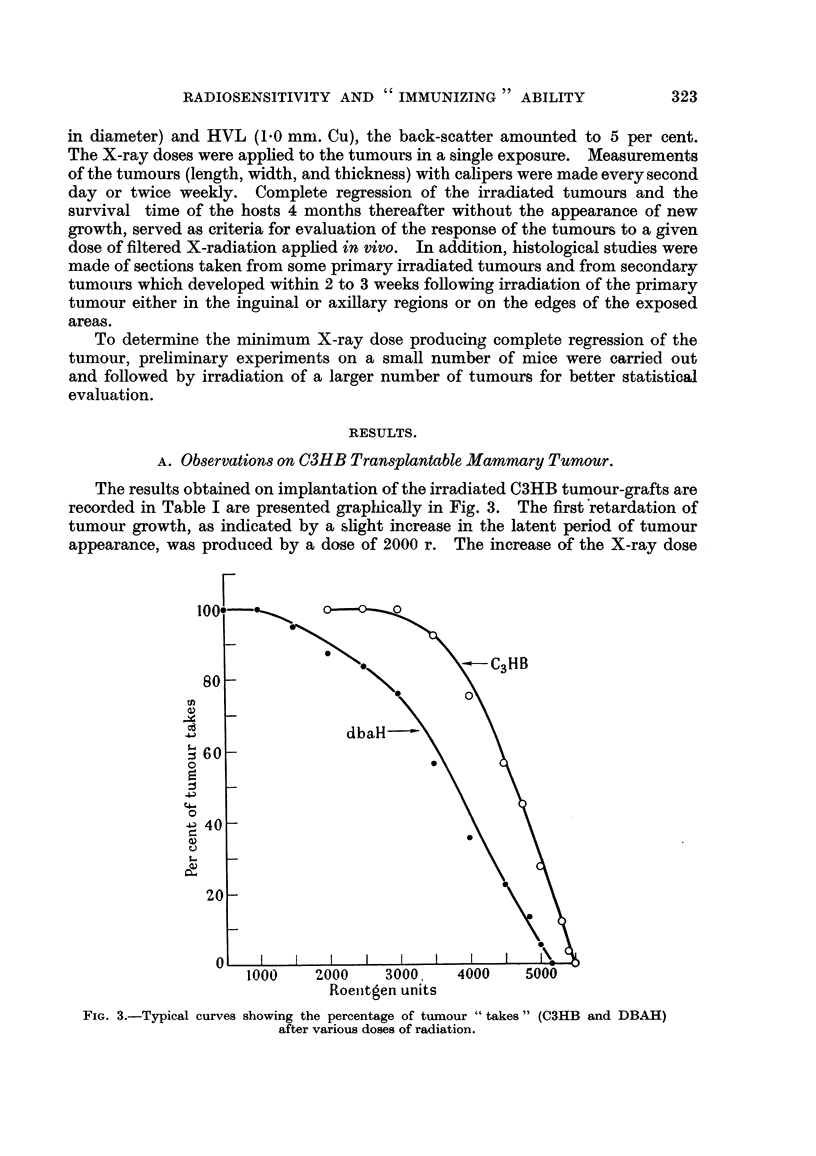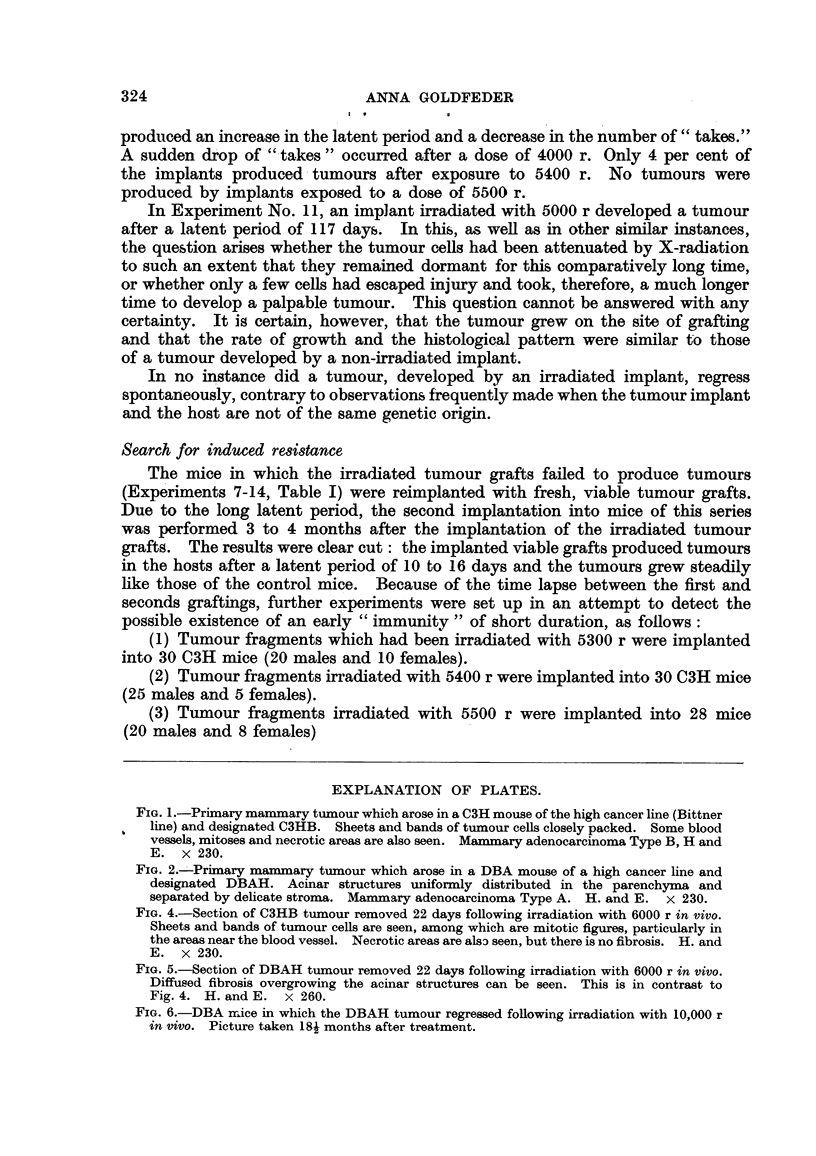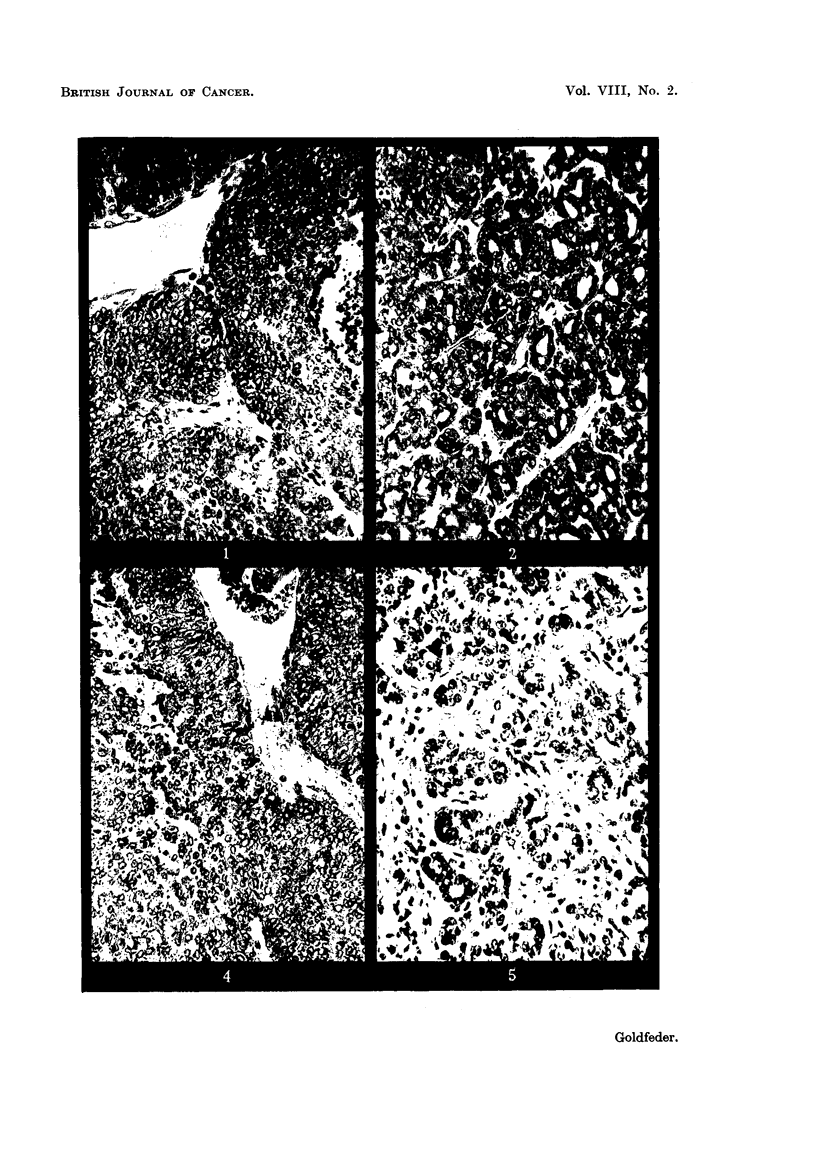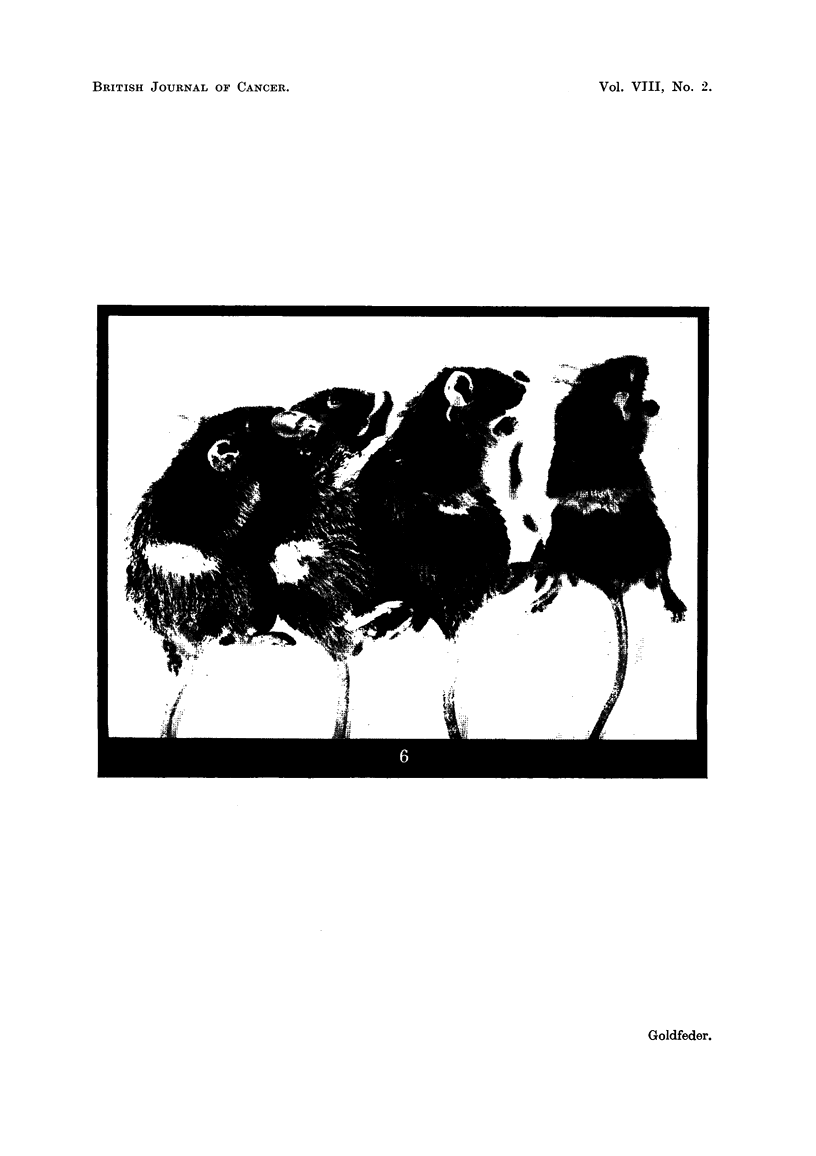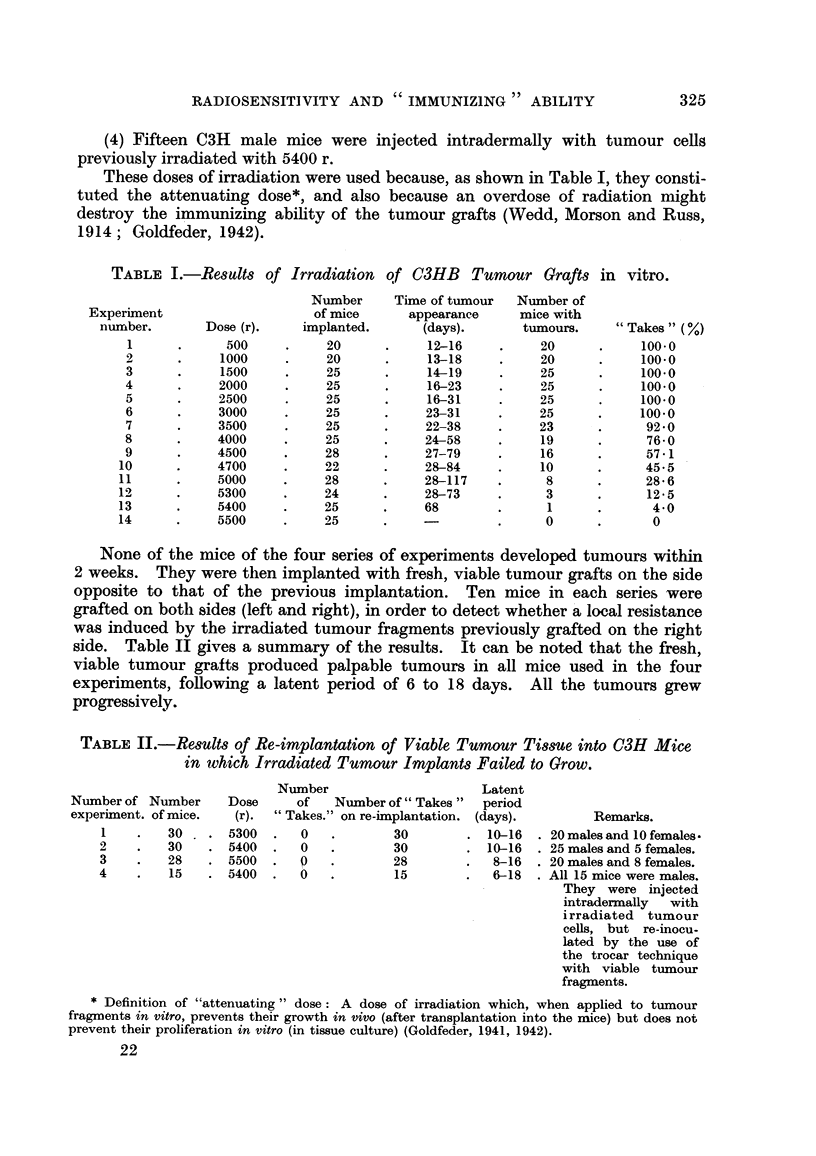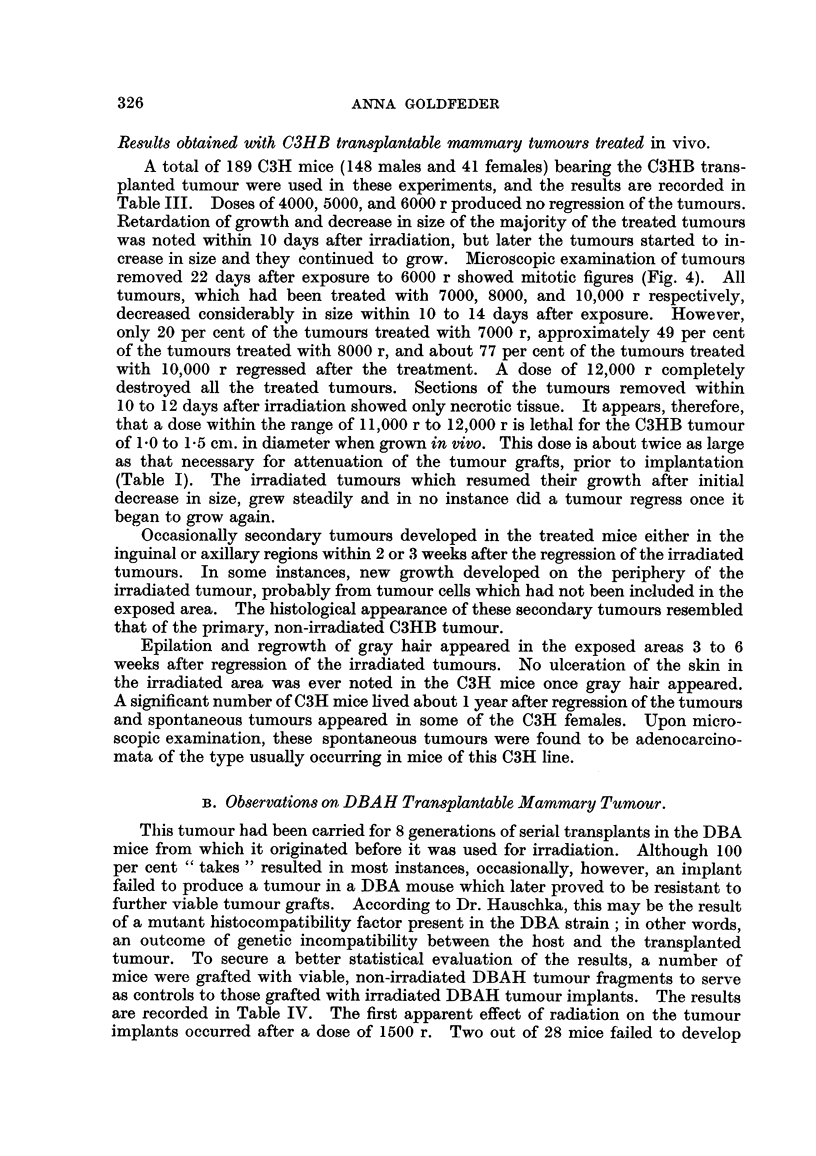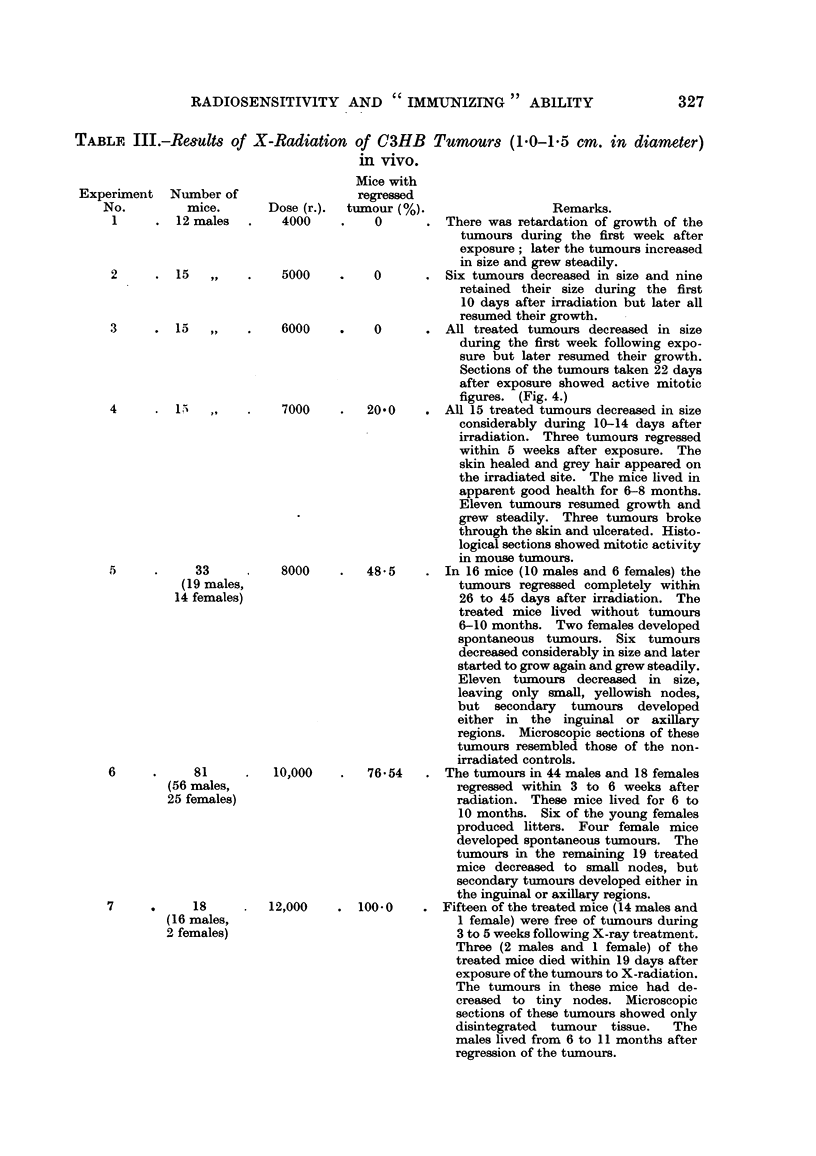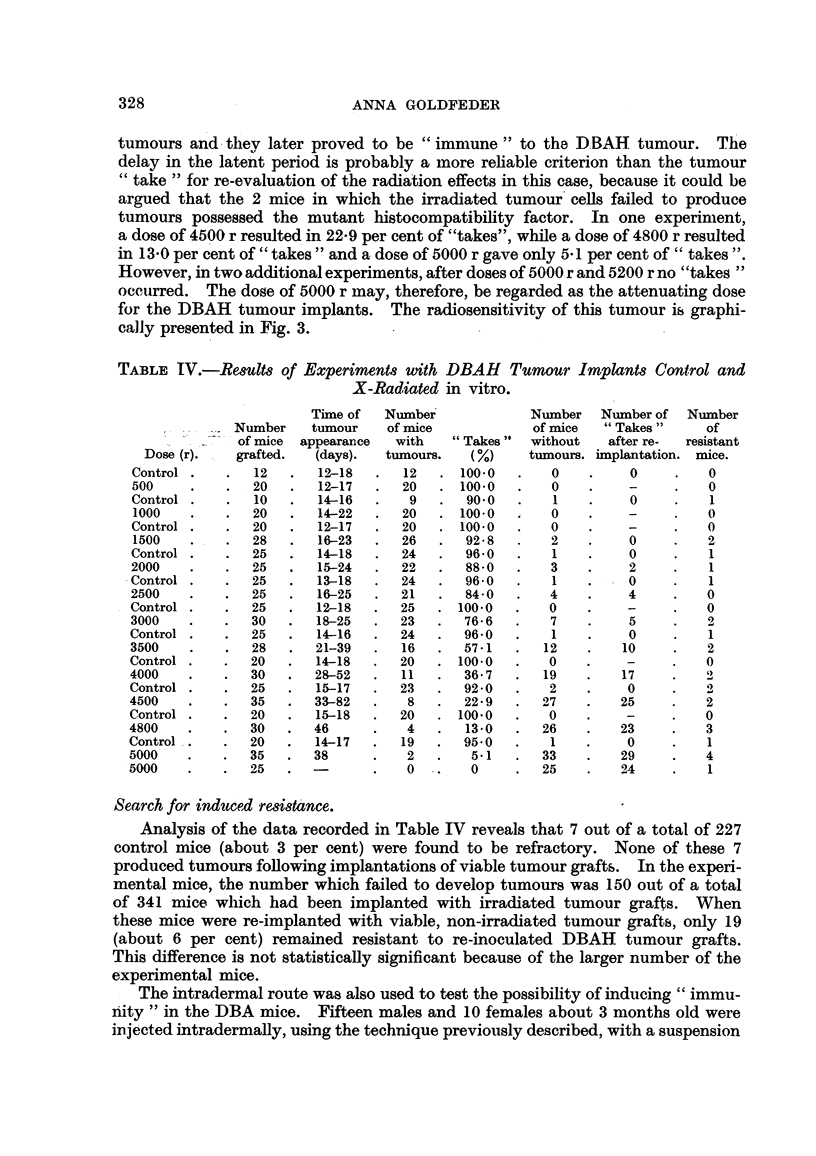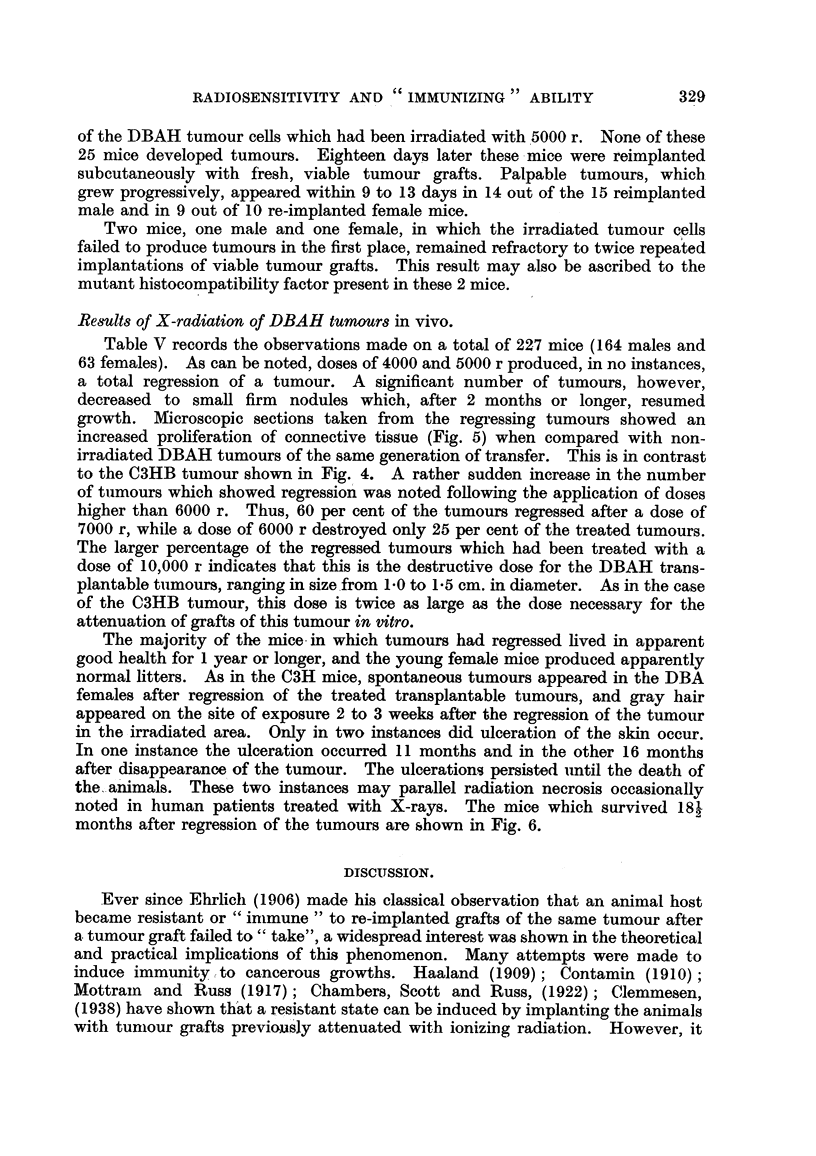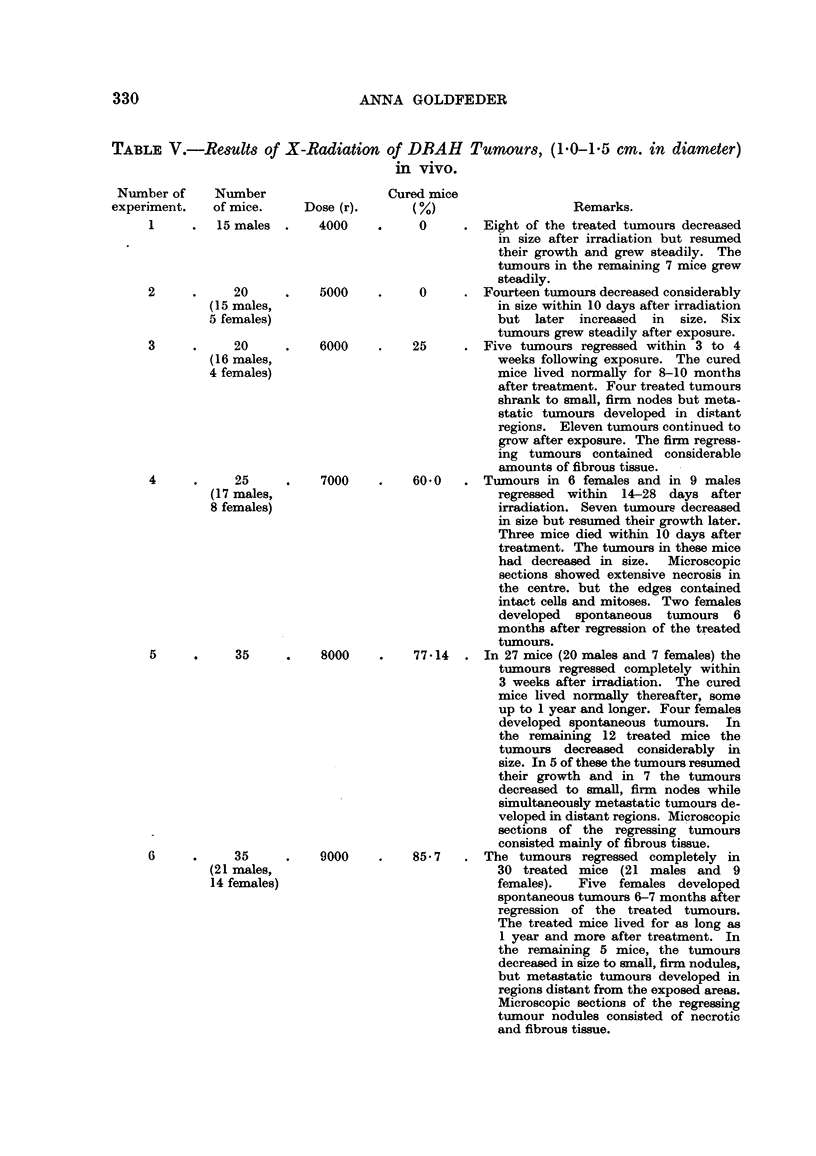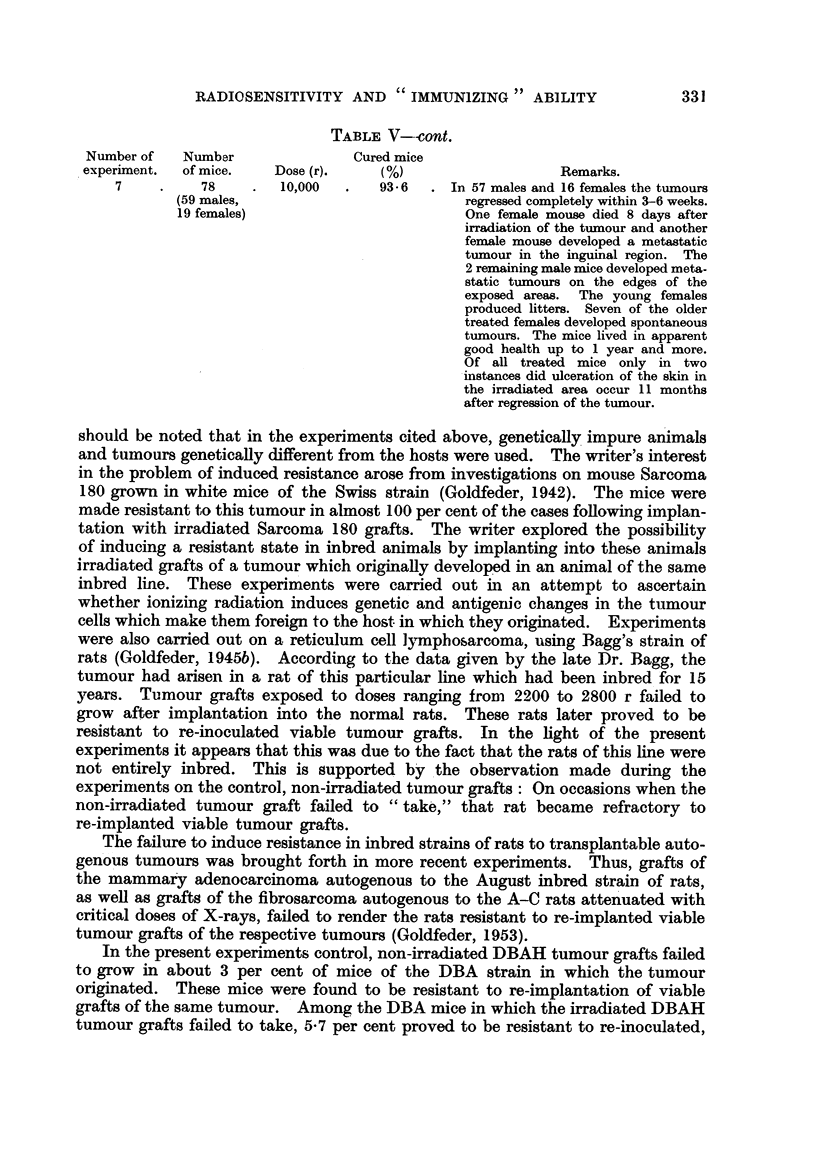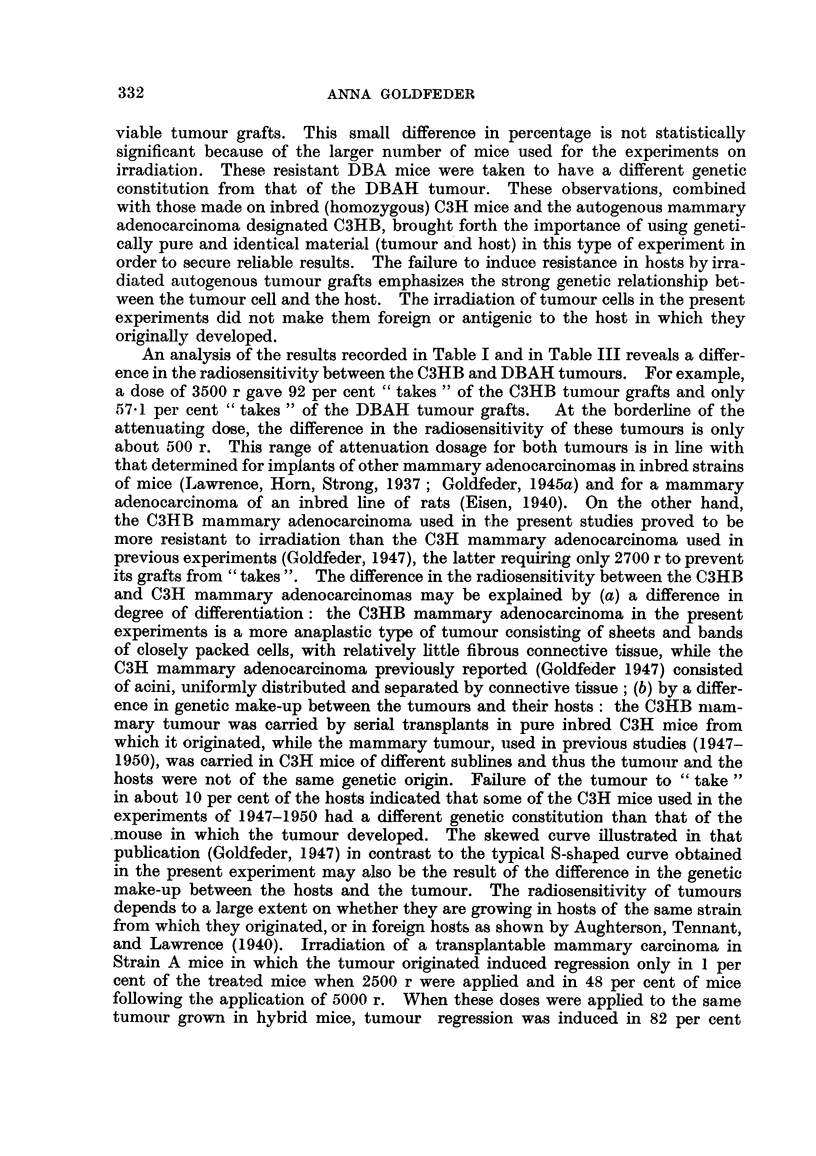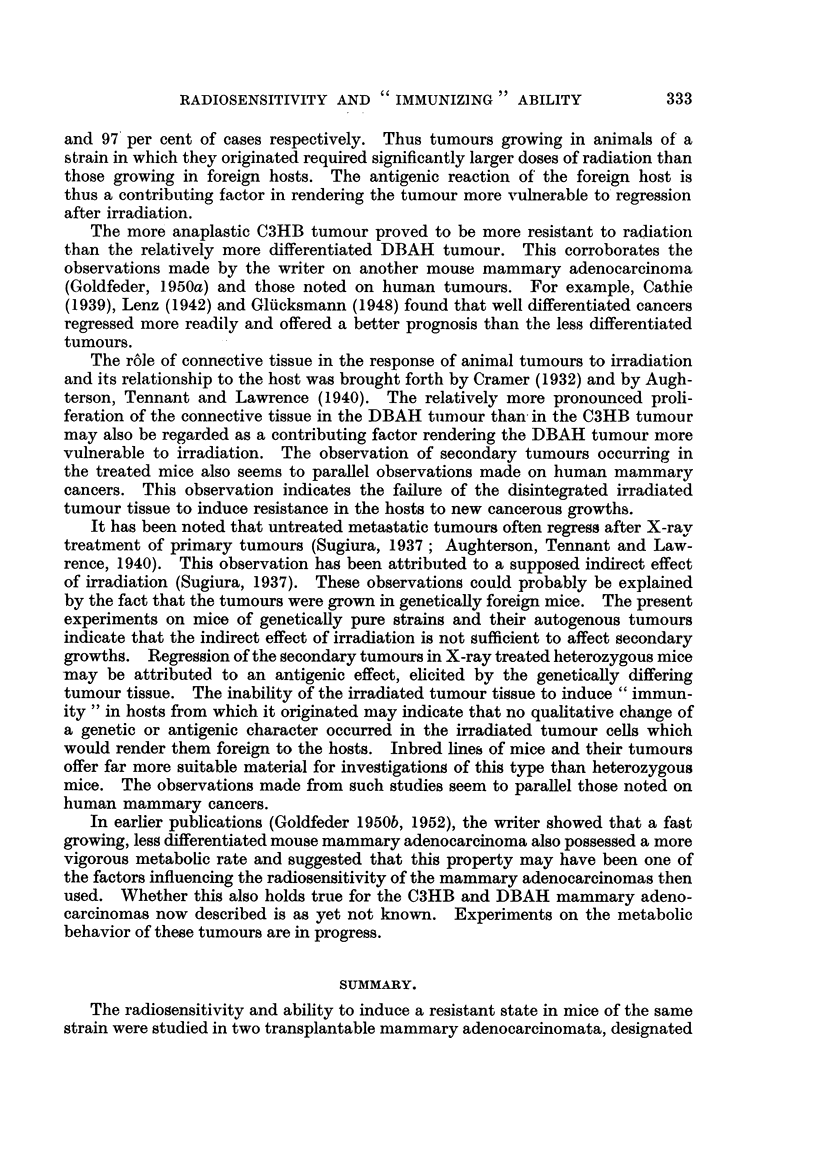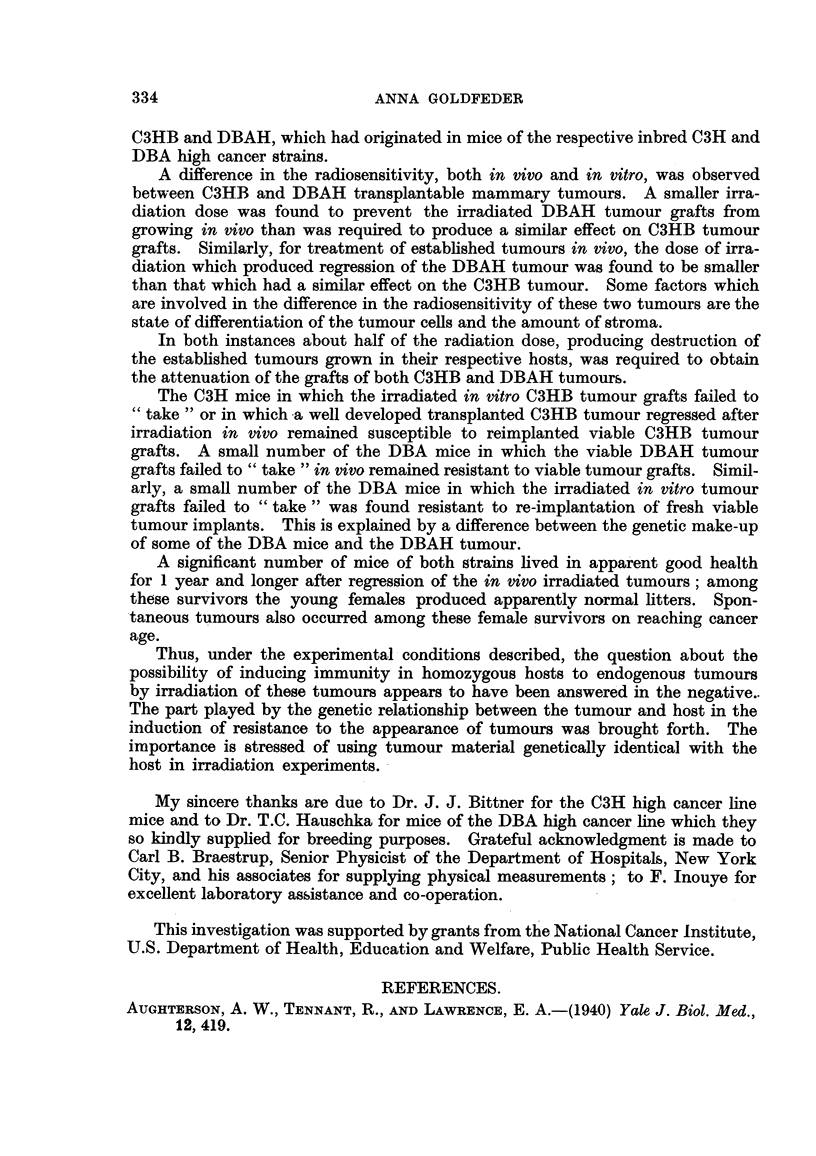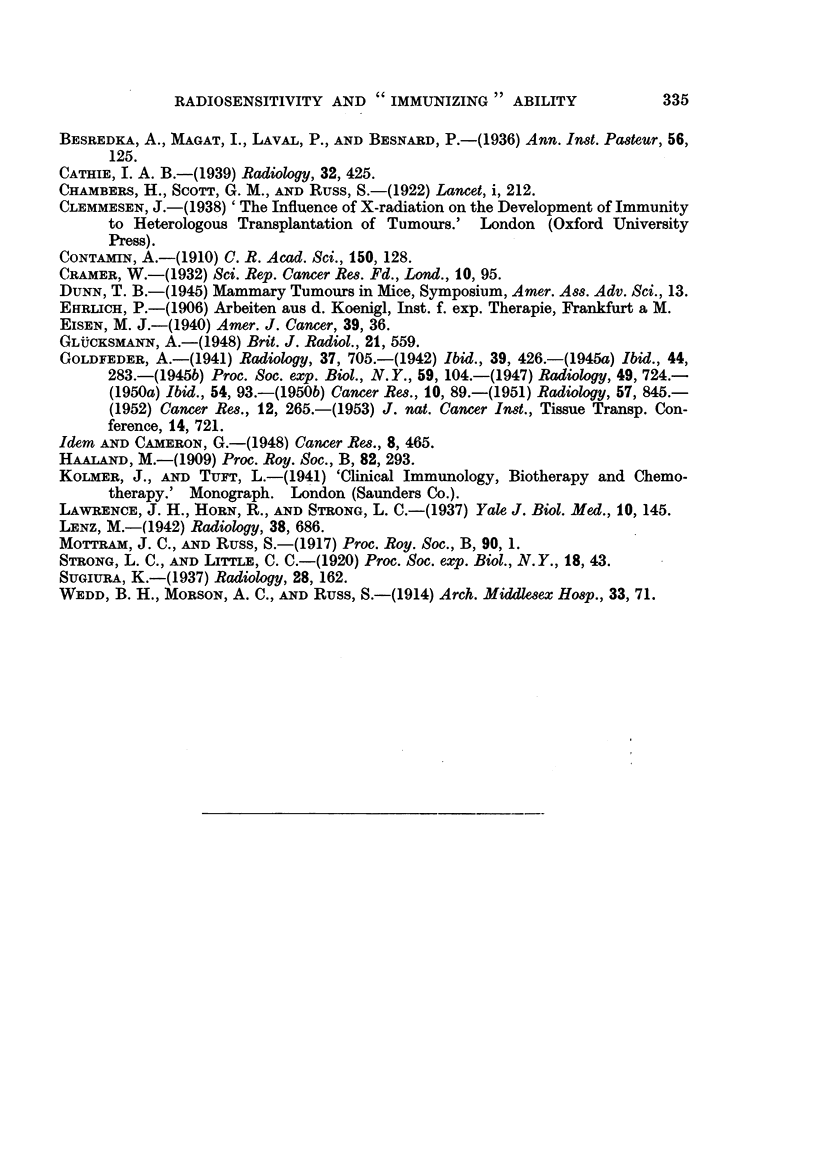# Studies on Radiosensitivity and “Immunizing” Ability of Mammary Tumours of Mice*

**DOI:** 10.1038/bjc.1954.33

**Published:** 1954-06

**Authors:** Anna Goldfeder

## Abstract

**Images:**


### STUDIES ON RADIOSENSITIVITY AND "IMMUNIZING" ABILITY OF MAMMARY TUMOURS OF MICE.*

ANNA GOLDFEDER.

*From the Cancer Research Laboratory, Department of Hospitals, City of New York, and the Department of Biology, Graduate School of Arts and Sciences, New York University.*

Received for publication April 5, 1954.

IN a series of earlier investigations it was shown that two transplantable mammary adenocarcinomata, one which arose in a mouse of the C3H strain and the other in a mouse of the DBA strain, differed significantly in their biological and physiological properties, as evidenced by their rate of growth, metabolic activity, and radiosensitivity (Goldfeder, 1947 ; Goldfeder and Cameron, 1948 ; Goldfeder, 1950*a*, 1950*b*, 1951). A significant number of mice of both C3H and DBA strains, in which either previously irradiated tumour grafts failed to produce tumours or established, actively growing tumours regressed following irradiation, became resistant to viable grafts of the respective tumours (Goldfeder, 1950*a*). However, it was not known whether these two tumours had been serially transplanted in mice genetically identical with those in which the tumours originally developed, as the mice were obtained from various laboratories (Goldfeder, 1950*a*). The mice used for transplantation undoubtedly came from different sublines of these two strains. Therefore, additional experiments were carried out in an attempt to elucidate the following questions :

1. What is the underlying factor or factors responsible for the difference in the radiosensitivity of mammary tumours?
2. Is it possible to induce an immune state in homozygous mice to autogenous tumours by inoculation of these tumours attenuated by irradiation?
3. Does the regression of a well-developed transplanted tumour following irradiation *in vivo* render the animal resistant to the development of spontaneous tumours?

This study was made possible by the use of the irradiation technique (Goldfeder, 1950*a*) which facilitates the exposure of the tumour and protects the rest of the body of the animal from secondary scattered radiation, thus permitting the treated animals to live their life span. Previous work on the irradiation of tumours has been summarized by the writer (1945*a*). There is no doubt that results obtained with tumours heterogenous to the animal hosts—that is, tumours which have a different genetic constitution from that of the host, are not comparable with those obtained with tumours autogenous to the animal hosts—that is with tumours having the same genetic constitution as that of the host, or with, those in patients suffering from cancer. As will be seen, the results obtained in animal experiments, using hosts and tumours of the same genetic derivation are comparable with observations frequently noted in human cancer patients.

* A preliminary report of this investigation was presented at the Annual Meeting of the American Association for Cancer Research, April, 1952, (Goldfeder, 1952).

### MATERIAL AND METHODS

C3H mice for breeding purposes were supplied by Dr. J. J. Bittner with the following information : " The mice we have sent you have been of pure lines tested to be homozygous ; they bear the milk agent and have an incidence of spontaneous tumours of over 90 per cent." Several pairs of DBA breeders were obtained from Dr. T. C. Hauschka, of the Cancer Institute in Philadelphia, who stated : " The Lankenau Hospital Research Institute originally obtained DBA mice from the Jackson B. Memorial Laboratory on July 5, 1933, and they were designated at that time as inbred strain ' D '. These mice have been inbred since : they behave immunologically like DBA/212, and have an incidence of over 85 per cent of spontaneous mammary tumours among breeding females. Mean tumour age 8 months ; range from 6–12 months ; based on a total of 62 breeding females."

Mice of both the C3H and DBA lines were bred by brother to sister matings in the writer's laboratory. Purina laboratory chow and water *ad libitum* constituted the basic diet. Mature mice also received lettuce or carrots twice weekly and sunflower seeds occasionally ; the nursing mothers received bread soaked in milk. Spontaneous tumours developed in 7–12-month-old females of both C3H and DBA strains.

A spontaneous mammary tumour of a 9-month-old C3H female mouse was excised under aseptic conditions. The edges of the tumour were cut with a sharp scalpel into pieces about 2 mm. in diameter and transplanted into 8 6-week-old mice. Palpable tumours appeared in all 8 mice within 14 to 16 days following transplantation.

Several cross-sections of the primary tumour were fixed in Zenker's fluid and stained with Harris haematoxylin and eosin. The histological appearance of the tumour was that of an adenocarcinoma Type B, according to the classification of Dunn (1945) (Fig. 1). This tumour has been carried in serial transplants in C3H mice for the past 4 years. There has been 100 per cent of " takes " and no instance of spontaneous regression. A tumour of about 5 mm. in diameter usually develops from an implant within a period of 12 to 14 days, and grows progressively.

A spontaneous mammary tumour of an 11-month-old female mouse of the DBA strain was excised aseptically. This tumour was firmer in consistency than that removed from the C3H mouse described. Fragments of about 2 mm. dissected from the edges of the tumour were implanted subcutaneously into 10 DBA mice (8 males and 2 females). Tumours of about 5 mm. in diameter appeared in all grafted mice within 16 to 18 days.

Cross-sections of the original tumour were fixed and stained by the same procedure as that used for the C3H tumour. This tumour according to Dunn (1945) represented an adenocarcinoma Type A (Fig. 2). It has been carried in serial transplants in mice of the DBA strain for more than 4 years. Its rate of growth is rather constant. Implants usually develop into tumours of measurable size within 12 to 16 days.

The spontaneous mammary tumours arising in mice of both strains (C3H and DBA) are of different types : Haemorrhagic, cystic, or solid, and varying in their degree of differentiation. Individual mammary tumours, not only in different mice of the same strain, but multiple tumours in the same mouse, may vary in their biological characteristics, as shown by Strong and Little (1920). Since the above-mentioned properties play a rôle in the radiosensitivity of tumours, the tumours used in the present experiments are therefore specifically designated C3HB and DBAH.

The investigation on the radiosensitivity of these tumours started after the eighth serial passage of both tumours.

The procedure of irradiation of tumour grafts *in vitro* was as follows : The edges of the tumour were cut into pieces of about 2 cm. in diameter which were then divided into two parts. One part was used for implantation into control mice, while the other was exposed to X-radiation. The tumour pieces used for irradiation were spread on a No. 1 round coverslip $\frac{7}{8}$ inch in diameter, which had been previously attached by a drop of sterile water to a mica sheet and covered with a Maximow depression slide, in the concavity of which moist filter paper had been placed to maintain the water content of the tissue. The tissue was exposed at 12·5 cm. target-tissue distance from an oil-cooled pulsating potential Coolidge tube. The radiation factors were : 200 kV, 20 mA, 0·5 mm. Cu plus 1·0 mm. Al filtration ; HVL of 1·1 mm. Cu. The dose rate was 602 r/min. measured in air ($\pm$ 5 per cent). The absorption of radiation by the cover glass, mica sheet and Maximow slide was about 10 per cent, which was taken into consideration in calculating the X-ray dose delivered to the tumour tissue. Immediately after irradiation the tumour fragments were implanted into about 2–3-month-old mice, most of which were litter mates. In two additional experiments the mice were injected intradermally with aliquots of irradiated tumour cells, suspended in physiological saline. This was done because, according to observations made by several investigators, the intradermal route of immunization appears to produce more effective and lasting results (Besredka, Magat, Laval and Besnard, 1936 ; Kolmar and Tuft, 1941). For the latter experiments the tumour fragments following irradiation were cut with sharp curved scissors until an almost homogeneous mass was obtained. A dense cell suspension was made in sterile 0·85 per cent saline solution (about 10,000 cells/c. mm.). This cell suspension was injected intracutaneously in 0·05 c.c. doses by means of a tuberculin syringe and 25 gauge needle in two or three sites of the abdominal wall of each mouse.

The latent period and the number of " takes " produced by tumour grafts irradiated *in vitro* served as criteria of the effect of a given dose of X-radiation.

The procedure of irradiation of the established tumours *in vivo* was as follows : Actively growing tumours ranging in size from 1·0 to 1·5 cm. in diameter were exposed to filtered X-radiation while the rest of the animal organism was shielded by a lead chamber (Goldfeder, 1950*a*). During the early experiments the tumours were irradiated *in vivo* under the same physical conditions as the tumour grafts *in vitro*. During the later experiments, the following physical factors were employed : The radiation was generated by a Westinghouse " Quadrocondex " Constant potential unit at 200 kV, 15 mA ; the incident beam was filtered by 0·5 mm. Cu plus 1·0 mm. Al ; HVL equalled 1·0 mm. Cu ; FSD, 20·0 cm. ; field size, 12 cm. in diameter. The dose rate measured in air by a thimble ionization chamber during the course of the investigations varied from 395 r/min. to 402 r/min. ($\pm$ 5 per cent). Special measurements were made with and without the lead shield, the ionization chamber being placed in the same position. The measurements indicated no significant scattering from the lead shield. Since the tumours were relatively much smaller than the field size they were exposed to a practically even distribution of radiation. Because of the sizes of the tumour (1·0 to 1·5 cm.

in diameter) and HVL (1·0 mm. Cu), the back-scatter amounted to 5 per cent. The X-ray doses were applied to the tumours in a single exposure. Measurements of the tumours (length, width, and thickness) with calipers were made every second day or twice weekly. Complete regression of the irradiated tumours and the survival time of the hosts 4 months thereafter without the appearance of new growth, served as criteria for evaluation of the response of the tumours to a given dose of filtered X-radiation applied *in vivo*. In addition, histological studies were made of sections taken from some primary irradiated tumours and from secondary tumours which developed within 2 to 3 weeks following irradiation of the primary tumour either in the inguinal or axillary regions or on the edges of the exposed areas.

To determine the minimum X-ray dose producing complete regression of the tumour, preliminary experiments on a small number of mice were carried out and followed by irradiation of a larger number of tumours for better statistical evaluation.

### RESULTS.

### A. *Observations on C3HB Transplantable Mammary Tumour.*

The results obtained on implantation of the irradiated C3HB tumour-grafts are recorded in Table I are presented graphically in Fig. 3. The first retardation of tumour growth, as indicated by a slight increase in the latent period of tumour appearance, was produced by a dose of 2000 r. The increase of the X-ray dose

FIG. 3.—Typical curves showing the percentage of tumour "takes" (C3HB and DBAH) after various doses of radiation.

produced an increase in the latent period and a decrease in the number of " takes." A sudden drop of " takes " occurred after a dose of 4000 r. Only 4 per cent of the implants produced tumours after exposure to 5400 r. No tumours were produced by implants exposed to a dose of 5500 r.

In Experiment No. 11, an implant irradiated with 5000 r developed a tumour after a latent period of 117 days. In this, as well as in other similar instances, the question arises whether the tumour cells had been attenuated by X-radiation to such an extent that they remained dormant for this comparatively long time, or whether only a few cells had escaped injury and took, therefore, a much longer time to develop a palpable tumour. This question cannot be answered with any certainty. It is certain, however, that the tumour grew on the site of grafting and that the rate of growth and the histological pattern were similar to those of a tumour developed by a non-irradiated implant.

In no instance did a tumour, developed by an irradiated implant, regress spontaneously, contrary to observations frequently made when the tumour implant and the host are not of the same genetic origin.

*Search for induced resistance*

The mice in which the irradiated tumour grafts failed to produce tumours (Experiments 7-14, Table I) were reimplanted with fresh, viable tumour grafts. Due to the long latent period, the second implantation into mice of this series was performed 3 to 4 months after the implantation of the irradiated tumour grafts. The results were clear cut : the implanted viable grafts produced tumours in the hosts after a latent period of 10 to 16 days and the tumours grew steadily like those of the control mice. Because of the time lapse between the first and seconds graftings, further experiments were set up in an attempt to detect the possible existence of an early " immunity " of short duration, as follows :

(1) Tumour fragments which had been irradiated with 5300 r were implanted into 30 C3H mice (20 males and 10 females).

(2) Tumour fragments irradiated with 5400 r were implanted into 30 C3H mice (25 males and 5 females).

(3) Tumour fragments irradiated with 5500 r were implanted into 28 mice (20 males and 8 females)

---

EXPLANATION OF PLATES.

FIG. 1.—Primary mammary tumour which arose in a C3H mouse of the high cancer line (Bittner line) and designated C3HB. Sheets and bands of tumour cells closely packed. Some blood vessels, mitoses and necrotic areas are also seen. Mammary adenocarcinoma Type B, H and E. × 230.

FIG. 2.—Primary mammary tumour which arose in a DBA mouse of a high cancer line and designated DBAH. Acinar structures uniformly distributed in the parenchyma and separated by delicate stroma. Mammary adenocarcinoma Type A. H. and E. × 230.

FIG. 4.—Section of C3HB tumour removed 22 days following irradiation with 6000 r *in vivo*. Sheets and bands of tumour cells are seen, among which are mitotic figures, particularly in the areas near the blood vessel. Necrotic areas are also seen, but there is no fibrosis. H. and E. × 230.

FIG. 5.—Section of DBAH tumour removed 22 days following irradiation with 6000 r *in vivo*. Diffused fibrosis overgrowing the acinar structures can be seen. This is in contrast to Fig. 4. H. and E. × 260.

FIG. 6.—DBA mice in which the DBAH tumour regressed following irradiation with 10,000 r *in vivo*. Picture taken 18½ months after treatment.

Goldfeder.

6

Goldfeder.

(4) Fifteen C3H male mice were injected intradermally with tumour cells previously irradiated with 5400 r.

These doses of irradiation were used because, as shown in Table I, they constituted the attenuating dose*, and also because an overdose of radiation might destroy the immunizing ability of the tumour grafts (Wedd, Morson and Russ, 1914; Goldfeder, 1942).

TABLE I.—*Results of Irradiation of C3HB Tumour Grafts* in vitro.

| Experiment number. | Dose (r). | Number of mice implanted. | Time of tumour appearance (days). | Number of mice with tumours. | "Takes" (%) |
|---|---|---|---|---|---|
| 1 | 500 | 20 | 12–16 | 20 | 100·0 |
| 2 | 1000 | 20 | 13–18 | 20 | 100·0 |
| 3 | 1500 | 25 | 14–19 | 25 | 100·0 |
| 4 | 2000 | 25 | 16–23 | 25 | 100·0 |
| 5 | 2500 | 25 | 16–31 | 25 | 100·0 |
| 6 | 3000 | 25 | 23–31 | 25 | 100·0 |
| 7 | 3500 | 25 | 22–38 | 23 | 92·0 |
| 8 | 4000 | 25 | 24–58 | 19 | 76·0 |
| 9 | 4500 | 28 | 27–79 | 16 | 57·1 |
| 10 | 4700 | 22 | 28–84 | 10 | 45·5 |
| 11 | 5000 | 28 | 28–117 | 8 | 28·6 |
| 12 | 5300 | 24 | 28–73 | 3 | 12·5 |
| 13 | 5400 | 25 | 68 | 1 | 4·0 |
| 14 | 5500 | 25 | — | 0 | 0 |

None of the mice of the four series of experiments developed tumours within 2 weeks. They were then implanted with fresh, viable tumour grafts on the side opposite to that of the previous implantation. Ten mice in each series were grafted on both sides (left and right), in order to detect whether a local resistance was induced by the irradiated tumour fragments previously grafted on the right side. Table II gives a summary of the results. It can be noted that the fresh, viable tumour grafts produced palpable tumours in all mice used in the four experiments, following a latent period of 6 to 18 days. All the tumours grew progressively.

TABLE II.—*Results of Re-implantation of Viable Tumour Tissue into C3H Mice in which Irradiated Tumour Implants Failed to Grow.*

| Number of experiment. | Number of mice. | Dose (r). | Number of "Takes." | Number of "Takes" on re-implantation. | Latent period (days). | Remarks. |
|---|---|---|---|---|---|---|
| 1 | 30 | 5300 | 0 | 30 | 10–16 | 20 males and 10 females. |
| 2 | 30 | 5400 | 0 | 30 | 10–16 | 25 males and 5 females. |
| 3 | 28 | 5500 | 0 | 28 | 8–16 | 20 males and 8 females. |
| 4 | 15 | 5400 | 0 | 15 | 6–18 | All 15 mice were males. They were injected intradermally with irradiated tumour cells, but re-inoculated by the use of the trocar technique with viable tumour fragments. |

* Definition of "attenuating" dose: A dose of irradiation which, when applied to tumour fragments *in vitro*, prevents their growth *in vivo* (after transplantation into the mice) but does not prevent their proliferation *in vitro* (in tissue culture) (Goldfeder, 1941, 1942).

*Results obtained with C3HB transplantable mammary tumours treated* in vivo.

A total of 189 C3H mice (148 males and 41 females) bearing the C3HB transplanted tumour were used in these experiments, and the results are recorded in Table III. Doses of 4000, 5000, and 6000 r produced no regression of the tumours. Retardation of growth and decrease in size of the majority of the treated tumours was noted within 10 days after irradiation, but later the tumours started to increase in size and they continued to grow. Microscopic examination of tumours removed 22 days after exposure to 6000 r showed mitotic figures (Fig. 4). All tumours, which had been treated with 7000, 8000, and 10,000 r respectively, decreased considerably in size within 10 to 14 days after exposure. However, only 20 per cent of the tumours treated with 7000 r, approximately 49 per cent of the tumours treated with 8000 r, and about 77 per cent of the tumours treated with 10,000 r regressed after the treatment. A dose of 12,000 r completely destroyed all the treated tumours. Sections of the tumours removed within 10 to 12 days after irradiation showed only necrotic tissue. It appears, therefore, that a dose within the range of 11,000 r to 12,000 r is lethal for the C3HB tumour of 1·0 to 1·5 cm. in diameter when grown *in vivo*. This dose is about twice as large as that necessary for attenuation of the tumour grafts, prior to implantation (Table I). The irradiated tumours which resumed their growth after initial decrease in size, grew steadily and in no instance did a tumour regress once it began to grow again.

Occasionally secondary tumours developed in the treated mice either in the inguinal or axillary regions within 2 or 3 weeks after the regression of the irradiated tumours. In some instances, new growth developed on the periphery of the irradiated tumour, probably from tumour cells which had not been included in the exposed area. The histological appearance of these secondary tumours resembled that of the primary, non-irradiated C3HB tumour.

Epilation and regrowth of gray hair appeared in the exposed areas 3 to 6 weeks after regression of the irradiated tumours. No ulceration of the skin in the irradiated area was ever noted in the C3H mice once gray hair appeared. A significant number of C3H mice lived about 1 year after regression of the tumours and spontaneous tumours appeared in some of the C3H females. Upon microscopic examination, these spontaneous tumours were found to be adenocarcinomata of the type usually occurring in mice of this C3H line.

### B. *Observations on DBAH Transplantable Mammary Tumour.*

This tumour had been carried for 8 generations of serial transplants in the DBA mice from which it originated before it was used for irradiation. Although 100 per cent " takes " resulted in most instances, occasionally, however, an implant failed to produce a tumour in a DBA mouse which later proved to be resistant to further viable tumour grafts. According to Dr. Hauschka, this may be the result of a mutant histocompatibility factor present in the DBA strain ; in other words, an outcome of genetic incompatibility between the host and the transplanted tumour. To secure a better statistical evaluation of the results, a number of mice were grafted with viable, non-irradiated DBAH tumour fragments to serve as controls to those grafted with irradiated DBAH tumour implants. The results are recorded in Table IV. The first apparent effect of radiation on the tumour implants occurred after a dose of 1500 r. Two out of 28 mice failed to develop

TABLE III.—*Results of X-Radiation of C3HB Tumours* (1·0–1·5 *cm. in diameter*) in vivo.

| Experiment No. | Number of mice. | Dose (r.). | Mice with regressed tumour (%). | Remarks. |
|---|---|---|---|---|
| 1 | 12 males | 4000 | 0 | There was retardation of growth of the tumours during the first week after exposure; later the tumours increased in size and grew steadily. |
| 2 | 15 ,, | 5000 | 0 | Six tumours decreased in size and nine retained their size during the first 10 days after irradiation but later all resumed their growth. |
| 3 | 15 ,, | 6000 | 0 | All treated tumours decreased in size during the first week following exposure but later resumed their growth. Sections of the tumours taken 22 days after exposure showed active mitotic figures. (Fig. 4.) |
| 4 | 15 ,, | 7000 | 20·0 | All 15 treated tumours decreased in size considerably during 10–14 days after irradiation. Three tumours regressed within 5 weeks after exposure. The skin healed and grey hair appeared on the irradiated site. The mice lived in apparent good health for 6–8 months. Eleven tumours resumed growth and grew steadily. Three tumours broke through the skin and ulcerated. Histological sections showed mitotic activity in mouse tumours. |
| 5 | 33 (19 males, 14 females) | 8000 | 48·5 | In 16 mice (10 males and 6 females) the tumours regressed completely within 26 to 45 days after irradiation. The treated mice lived without tumours 6–10 months. Two females developed spontaneous tumours. Six tumours decreased considerably in size and later started to grow again and grew steadily. Eleven tumours decreased in size, leaving only small, yellowish nodes, but secondary tumours developed either in the inguinal or axillary regions. Microscopic sections of these tumours resembled those of the non-irradiated controls. |
| 6 | 81 (56 males, 25 females) | 10,000 | 76·54 | The tumours in 44 males and 18 females regressed within 3 to 6 weeks after radiation. These mice lived for 6 to 10 months. Six of the young females produced litters. Four female mice developed spontaneous tumours. The tumours in the remaining 19 treated mice decreased to small nodes, but secondary tumours developed either in the inguinal or axillary regions. |
| 7 | 18 (16 males, 2 females) | 12,000 | 100·0 | Fifteen of the treated mice (14 males and 1 female) were free of tumours during 3 to 5 weeks following X-ray treatment. Three (2 males and 1 female) of the treated mice died within 19 days after exposure of the tumours to X-radiation. The tumours in these mice had decreased to tiny nodes. Microscopic sections of these tumours showed only disintegrated tumour tissue. The males lived from 6 to 11 months after regression of the tumours. |

tumours and they later proved to be " immune " to the DBAH tumour. The delay in the latent period is probably a more reliable criterion than the tumour " take " for re-evaluation of the radiation effects in this case, because it could be argued that the 2 mice in which the irradiated tumour cells failed to produce tumours possessed the mutant histocompatibility factor. In one experiment, a dose of 4500 r resulted in 22·9 per cent of "takes", while a dose of 4800 r resulted in 13·0 per cent of " takes " and a dose of 5000 r gave only 5·1 per cent of " takes ". However, in two additional experiments, after doses of 5000 r and 5200 r no "takes " occurred. The dose of 5000 r may, therefore, be regarded as the attenuating dose for the DBAH tumour implants. The radiosensitivity of this tumour is graphically presented in Fig. 3.

TABLE IV.—*Results of Experiments with DBAH Tumour Implants Control and X-Radiated* in vitro.

| Dose (r). | Number of mice grafted. | Time of tumour appearance (days). | Number of mice with tumours. | " Takes " (%) | Number of mice without tumours. | Number of " Takes " after re-implantation. | Number of resistant mice. |
|---|---|---|---|---|---|---|---|
| Control | 12 | 12–18 | 12 | 100·0 | 0 | 0 | 0 |
| 500 | 20 | 12–17 | 20 | 100·0 | 0 | – | 0 |
| Control | 10 | 14–16 | 9 | 90·0 | 1 | 0 | 1 |
| 1000 | 20 | 14–22 | 20 | 100·0 | 0 | – | 0 |
| Control | 20 | 12–17 | 20 | 100·0 | 0 | – | 0 |
| 1500 | 28 | 16–23 | 26 | 92·8 | 2 | 0 | 2 |
| Control | 25 | 14–18 | 24 | 96·0 | 1 | 0 | 1 |
| 2000 | 25 | 15–24 | 22 | 88·0 | 3 | 2 | 1 |
| Control | 25 | 13–18 | 24 | 96·0 | 1 | 0 | 1 |
| 2500 | 25 | 16–25 | 21 | 84·0 | 4 | 4 | 0 |
| Control | 25 | 12–18 | 25 | 100·0 | 0 | – | 0 |
| 3000 | 30 | 18–25 | 23 | 76·6 | 7 | 5 | 2 |
| Control | 25 | 14–16 | 24 | 96·0 | 1 | 0 | 1 |
| 3500 | 28 | 21–39 | 16 | 57·1 | 12 | 10 | 2 |
| Control | 20 | 14–18 | 20 | 100·0 | 0 | – | 0 |
| 4000 | 30 | 28–52 | 11 | 36·7 | 19 | 17 | 2 |
| Control | 25 | 15–17 | 23 | 92·0 | 2 | 0 | 2 |
| 4500 | 35 | 33–82 | 8 | 22·9 | 27 | 25 | 2 |
| Control | 20 | 15–18 | 20 | 100·0 | 0 | – | 0 |
| 4800 | 30 | 46 | 4 | 13·0 | 26 | 23 | 3 |
| Control | 20 | 14–17 | 19 | 95·0 | 1 | 0 | 1 |
| 5000 | 35 | 38 | 2 | 5·1 | 33 | 29 | 4 |
| 5000 | 25 | — | 0 | 0 | 25 | 24 | 1 |

*Search for induced resistance.*

Analysis of the data recorded in Table IV reveals that 7 out of a total of 227 control mice (about 3 per cent) were found to be refractory. None of these 7 produced tumours following implantations of viable tumour grafts. In the experimental mice, the number which failed to develop tumours was 150 out of a total of 341 mice which had been implanted with irradiated tumour grafts. When these mice were re-implanted with viable, non-irradiated tumour grafts, only 19 (about 6 per cent) remained resistant to re-inoculated DBAH tumour grafts. This difference is not statistically significant because of the larger number of the experimental mice.

The intradermal route was also used to test the possibility of inducing " immunity " in the DBA mice. Fifteen males and 10 females about 3 months old were injected intradermally, using the technique previously described, with a suspension of the DBAH tumour cells which had been irradiated with 5000 r. None of these 25 mice developed tumours. Eighteen days later these mice were reimplanted subcutaneously with fresh, viable tumour grafts. Palpable tumours, which grew progressively, appeared within 9 to 13 days in 14 out of the 15 reimplanted male and in 9 out of 10 re-implanted female mice.

Two mice, one male and one female, in which the irradiated tumour cells failed to produce tumours in the first place, remained refractory to twice repeated implantations of viable tumour grafts. This result may also be ascribed to the mutant histocompatibility factor present in these 2 mice.

*Results of X-radiation of DBAH tumours* in vivo.

Table V records the observations made on a total of 227 mice (164 males and 63 females). As can be noted, doses of 4000 and 5000 r produced, in no instances, a total regression of a tumour. A significant number of tumours, however, decreased to small firm nodules which, after 2 months or longer, resumed growth. Microscopic sections taken from the regressing tumours showed an increased proliferation of connective tissue (Fig. 5) when compared with non-irradiated DBAH tumours of the same generation of transfer. This is in contrast to the C3HB tumour shown in Fig. 4. A rather sudden increase in the number of tumours which showed regression was noted following the application of doses higher than 6000 r. Thus, 60 per cent of the tumours regressed after a dose of 7000 r, while a dose of 6000 r destroyed only 25 per cent of the treated tumours. The larger percentage of the regressed tumours which had been treated with a dose of 10,000 r indicates that this is the destructive dose for the DBAH transplantable tumours, ranging in size from 1·0 to 1·5 cm. in diameter. As in the case of the C3HB tumour, this dose is twice as large as the dose necessary for the attenuation of grafts of this tumour *in vitro*.

The majority of the mice in which tumours had regressed lived in apparent good health for 1 year or longer, and the young female mice produced apparently normal litters. As in the C3H mice, spontaneous tumours appeared in the DBA females after regression of the treated transplantable tumours, and gray hair appeared on the site of exposure 2 to 3 weeks after the regression of the tumour in the irradiated area. Only in two instances did ulceration of the skin occur. In one instance the ulceration occurred 11 months and in the other 16 months after disappearance of the tumour. The ulcerations persisted until the death of the animals. These two instances may parallel radiation necrosis occasionally noted in human patients treated with X-rays. The mice which survived 18½ months after regression of the tumours are shown in Fig. 6.

### DISCUSSION.

Ever since Ehrlich (1906) made his classical observation that an animal host became resistant or "immune" to re-implanted grafts of the same tumour after a tumour graft failed to "take", a widespread interest was shown in the theoretical and practical implications of this phenomenon. Many attempts were made to induce immunity to cancerous growths. Haaland (1909); Contamin (1910); Mottram and Russ (1917); Chambers, Scott and Russ, (1922); Clemmesen, (1938) have shown that a resistant state can be induced by implanting the animals with tumour grafts previously attenuated with ionizing radiation. However, it

TABLE V.—*Results of X-Radiation of DBAH Tumours,* (1·0–1·5 *cm. in diameter*) in vivo.

| Number of experiment. | Number of mice. | Dose (r). | Cured mice (%) | Remarks. |
|---|---|---|---|---|
| 1 | 15 males | 4000 | 0 | Eight of the treated tumours decreased in size after irradiation but resumed their growth and grew steadily. The tumours in the remaining 7 mice grew steadily. |
| 2 | 20 (15 males, 5 females) | 5000 | 0 | Fourteen tumours decreased considerably in size within 10 days after irradiation but later increased in size. Six tumours grew steadily after exposure. |
| 3 | 20 (16 males, 4 females) | 6000 | 25 | Five tumours regressed within 3 to 4 weeks following exposure. The cured mice lived normally for 8–10 months after treatment. Four treated tumours shrank to small, firm nodes but metastatic tumours developed in distant regions. Eleven tumours continued to grow after exposure. The firm regressing tumours contained considerable amounts of fibrous tissue. |
| 4 | 25 (17 males, 8 females) | 7000 | 60·0 | Tumours in 6 females and in 9 males regressed within 14–28 days after irradiation. Seven tumours decreased in size but resumed their growth later. Three mice died within 10 days after treatment. The tumours in these mice had decreased in size. Microscopic sections showed extensive necrosis in the centre. but the edges contained intact cells and mitoses. Two females developed spontaneous tumours 6 months after regression of the treated tumours. |
| 5 | 35 | 8000 | 77·14 | In 27 mice (20 males and 7 females) the tumours regressed completely within 3 weeks after irradiation. The cured mice lived normally thereafter, some up to 1 year and longer. Four females developed spontaneous tumours. In the remaining 12 treated mice the tumours decreased considerably in size. In 5 of these the tumours resumed their growth and in 7 the tumours decreased to small, firm nodes while simultaneously metastatic tumours developed in distant regions. Microscopic sections of the regressing tumours consisted mainly of fibrous tissue. |
| 6 | 35 (21 males, 14 females) | 9000 | 85·7 | The tumours regressed completely in 30 treated mice (21 males and 9 females). Five females developed spontaneous tumours 6–7 months after regression of the treated tumours. The treated mice lived for as long as 1 year and more after treatment. In the remaining 5 mice, the tumours decreased in size to small, firm nodules, but metastatic tumours developed in regions distant from the exposed areas. Microscopic sections of the regressing tumour nodules consisted of necrotic and fibrous tissue. |

TABLE V—*cont.*

| Number of experiment. | Number of mice. | Dose (r). | Cured mice (%) | Remarks. |
|---|---|---|---|---|
| 7 | 78 (59 males, 19 females) | 10,000 | 93·6 | In 57 males and 16 females the tumours regressed completely within 3–6 weeks. One female mouse died 8 days after irradiation of the tumour and another female mouse developed a metastatic tumour in the inguinal region. The 2 remaining male mice developed metastatic tumours on the edges of the exposed areas. The young females produced litters. Seven of the older treated females developed spontaneous tumours. The mice lived in apparent good health up to 1 year and more. Of all treated mice only in two instances did ulceration of the skin in the irradiated area occur 11 months after regression of the tumour. |

should be noted that in the experiments cited above, genetically impure animals and tumours genetically different from the hosts were used. The writer's interest in the problem of induced resistance arose from investigations on mouse Sarcoma 180 grown in white mice of the Swiss strain (Goldfeder, 1942). The mice were made resistant to this tumour in almost 100 per cent of the cases following implantation with irradiated Sarcoma 180 grafts. The writer explored the possibility of inducing a resistant state in inbred animals by implanting into these animals irradiated grafts of a tumour which originally developed in an animal of the same inbred line. These experiments were carried out in an attempt to ascertain whether ionizing radiation induces genetic and antigenic changes in the tumour cells which make them foreign to the host in which they originated. Experiments were also carried out on a reticulum cell lymphosarcoma, using Bagg's strain of rats (Goldfeder, 1945*b*). According to the data given by the late Dr. Bagg, the tumour had arisen in a rat of this particular line which had been inbred for 15 years. Tumour grafts exposed to doses ranging from 2200 to 2800 r failed to grow after implantation into the normal rats. These rats later proved to be resistant to re-inoculated viable tumour grafts. In the light of the present experiments it appears that this was due to the fact that the rats of this line were not entirely inbred. This is supported by the observation made during the experiments on the control, non-irradiated tumour grafts : On occasions when the non-irradiated tumour graft failed to "take," that rat became refractory to re-implanted viable tumour grafts.

The failure to induce resistance in inbred strains of rats to transplantable autogenous tumours was brought forth in more recent experiments. Thus, grafts of the mammary adenocarcinoma autogenous to the August inbred strain of rats, as well as grafts of the fibrosarcoma autogenous to the A–C rats attenuated with critical doses of X-rays, failed to render the rats resistant to re-implanted viable tumour grafts of the respective tumours (Goldfeder, 1953).

In the present experiments control, non-irradiated DBAH tumour grafts failed to grow in about 3 per cent of mice of the DBA strain in which the tumour originated. These mice were found to be resistant to re-implantation of viable grafts of the same tumour. Among the DBA mice in which the irradiated DBAH tumour grafts failed to take, 5·7 per cent proved to be resistant to re-inoculated, viable tumour grafts. This small difference in percentage is not statistically significant because of the larger number of mice used for the experiments on irradiation. These resistant DBA mice were taken to have a different genetic constitution from that of the DBAH tumour. These observations, combined with those made on inbred (homozygous) C3H mice and the autogenous mammary adenocarcinoma designated C3HB, brought forth the importance of using genetically pure and identical material (tumour and host) in this type of experiment in order to secure reliable results. The failure to induce resistance in hosts by irradiated autogenous tumour grafts emphasizes the strong genetic relationship between the tumour cell and the host. The irradiation of tumour cells in the present experiments did not make them foreign or antigenic to the host in which they originally developed.

An analysis of the results recorded in Table I and in Table III reveals a difference in the radiosensitivity between the C3HB and DBAH tumours. For example, a dose of 3500 r gave 92 per cent " takes " of the C3HB tumour grafts and only 57·1 per cent " takes " of the DBAH tumour grafts. At the borderline of the attenuating dose, the difference in the radiosensitivity of these tumours is only about 500 r. This range of attenuation dosage for both tumours is in line with that determined for implants of other mammary adenocarcinomas in inbred strains of mice (Lawrence, Horn, Strong, 1937 ; Goldfeder, 1945*a*) and for a mammary adenocarcinoma of an inbred line of rats (Eisen, 1940). On the other hand, the C3HB mammary adenocarcinoma used in the present studies proved to be more resistant to irradiation than the C3H mammary adenocarcinoma used in previous experiments (Goldfeder, 1947), the latter requiring only 2700 r to prevent its grafts from " takes ". The difference in the radiosensitivity between the C3HB and C3H mammary adenocarcinomas may be explained by (*a*) a difference in degree of differentiation : the C3HB mammary adenocarcinoma in the present experiments is a more anaplastic type of tumour consisting of sheets and bands of closely packed cells, with relatively little fibrous connective tissue, while the C3H mammary adenocarcinoma previously reported (Goldfeder 1947) consisted of acini, uniformly distributed and separated by connective tissue ; (*b*) by a difference in genetic make-up between the tumours and their hosts : the C3HB mammary tumour was carried by serial transplants in pure inbred C3H mice from which it originated, while the mammary tumour, used in previous studies (1947–1950), was carried in C3H mice of different sublines and thus the tumour and the hosts were not of the same genetic origin. Failure of the tumour to " take " in about 10 per cent of the hosts indicated that some of the C3H mice used in the experiments of 1947–1950 had a different genetic constitution than that of the mouse in which the tumour developed. The skewed curve illustrated in that publication (Goldfeder, 1947) in contrast to the typical S-shaped curve obtained in the present experiment may also be the result of the difference in the genetic make-up between the hosts and the tumour. The radiosensitivity of tumours depends to a large extent on whether they are growing in hosts of the same strain from which they originated, or in foreign hosts as shown by Aughterson, Tennant, and Lawrence (1940). Irradiation of a transplantable mammary carcinoma in Strain A mice in which the tumour originated induced regression only in 1 per cent of the treated mice when 2500 r were applied and in 48 per cent of mice following the application of 5000 r. When these doses were applied to the same tumour grown in hybrid mice, tumour regression was induced in 82 per cent and 97 per cent of cases respectively. Thus tumours growing in animals of a strain in which they originated required significantly larger doses of radiation than those growing in foreign hosts. The antigenic reaction of the foreign host is thus a contributing factor in rendering the tumour more vulnerable to regression after irradiation.

The more anaplastic C3HB tumour proved to be more resistant to radiation than the relatively more differentiated DBAH tumour. This corroborates the observations made by the writer on another mouse mammary adenocarcinoma (Goldfeder, 1950*a*) and those noted on human tumours. For example, Cathie (1939), Lenz (1942) and Glücksmann (1948) found that well differentiated cancers regressed more readily and offered a better prognosis than the less differentiated tumours.

The rôle of connective tissue in the response of animal tumours to irradiation and its relationship to the host was brought forth by Cramer (1932) and by Aughterson, Tennant and Lawrence (1940). The relatively more pronounced proliferation of the connective tissue in the DBAH tumour than in the C3HB tumour may also be regarded as a contributing factor rendering the DBAH tumour more vulnerable to irradiation. The observation of secondary tumours occurring in the treated mice also seems to parallel observations made on human mammary cancers. This observation indicates the failure of the disintegrated irradiated tumour tissue to induce resistance in the hosts to new cancerous growths.

It has been noted that untreated metastatic tumours often regress after X-ray treatment of primary tumours (Sugiura, 1937 ; Aughterson, Tennant and Lawrence, 1940). This observation has been attributed to a supposed indirect effect of irradiation (Sugiura, 1937). These observations could probably be explained by the fact that the tumours were grown in genetically foreign mice. The present experiments on mice of genetically pure strains and their autogenous tumours indicate that the indirect effect of irradiation is not sufficient to affect secondary growths. Regression of the secondary tumours in X-ray treated heterozygous mice may be attributed to an antigenic effect, elicited by the genetically differing tumour tissue. The inability of the irradiated tumour tissue to induce " immunity " in hosts from which it originated may indicate that no qualitative change of a genetic or antigenic character occurred in the irradiated tumour cells which would render them foreign to the hosts. Inbred lines of mice and their tumours offer far more suitable material for investigations of this type than heterozygous mice. The observations made from such studies seem to parallel those noted on human mammary cancers.

In earlier publications (Goldfeder 1950*b*, 1952), the writer showed that a fast growing, less differentiated mouse mammary adenocarcinoma also possessed a more vigorous metabolic rate and suggested that this property may have been one of the factors influencing the radiosensitivity of the mammary adenocarcinomas then used. Whether this also holds true for the C3HB and DBAH mammary adenocarcinomas now described is as yet not known. Experiments on the metabolic behavior of these tumours are in progress.

### SUMMARY.

The radiosensitivity and ability to induce a resistant state in mice of the same strain were studied in two transplantable mammary adenocarcinomata, designated

C3HB and DBAH, which had originated in mice of the respective inbred C3H and DBA high cancer strains.

A difference in the radiosensitivity, both *in vivo* and *in vitro*, was observed between C3HB and DBAH transplantable mammary tumours. A smaller irradiation dose was found to prevent the irradiated DBAH tumour grafts from growing *in vivo* than was required to produce a similar effect on C3HB tumour grafts. Similarly, for treatment of established tumours *in vivo*, the dose of irradiation which produced regression of the DBAH tumour was found to be smaller than that which had a similar effect on the C3HB tumour. Some factors which are involved in the difference in the radiosensitivity of these two tumours are the state of differentiation of the tumour cells and the amount of stroma.

In both instances about half of the radiation dose, producing destruction of the established tumours grown in their respective hosts, was required to obtain the attenuation of the grafts of both C3HB and DBAH tumours.

The C3H mice in which the irradiated *in vitro* C3HB tumour grafts failed to " take " or in which a well developed transplanted C3HB tumour regressed after irradiation *in vivo* remained susceptible to reimplanted viable C3HB tumour grafts. A small number of the DBA mice in which the viable DBAH tumour grafts failed to " take " *in vivo* remained resistant to viable tumour grafts. Similarly, a small number of the DBA mice in which the irradiated *in vitro* tumour grafts failed to " take " was found resistant to re-implantation of fresh viable tumour implants. This is explained by a difference between the genetic make-up of some of the DBA mice and the DBAH tumour.

A significant number of mice of both strains lived in apparent good health for 1 year and longer after regression of the *in vivo* irradiated tumours ; among these survivors the young females produced apparently normal litters. Spontaneous tumours also occurred among these female survivors on reaching cancer age.

Thus, under the experimental conditions described, the question about the possibility of inducing immunity in homozygous hosts to endogenous tumours by irradiation of these tumours appears to have been answered in the negative. The part played by the genetic relationship between the tumour and host in the induction of resistance to the appearance of tumours was brought forth. The importance is stressed of using tumour material genetically identical with the host in irradiation experiments.

My sincere thanks are due to Dr. J. J. Bittner for the C3H high cancer line mice and to Dr. T.C. Hauschka for mice of the DBA high cancer line which they so kindly supplied for breeding purposes. Grateful acknowledgment is made to Carl B. Braestrup, Senior Physicist of the Department of Hospitals, New York City, and his associates for supplying physical measurements ; to F. Inouye for excellent laboratory assistance and co-operation.

This investigation was supported by grants from the National Cancer Institute, U.S. Department of Health, Education and Welfare, Public Health Service.

### REFERENCES.

AUGHTERSON, A. W., TENNANT, R., AND LAWRENCE, E. A.—(1940) *Yale J. Biol. Med.*, **12**, 419.

BESREDKA, A., MAGAT, I., LAVAL, P., AND BESNARD, P.—(1936) *Ann. Inst. Pasteur*, **56**, 125.
CATHIE, I. A. B.—(1939) *Radiology*, **32**, 425.
CHAMBERS, H., SCOTT, G. M., AND RUSS, S.—(1922) *Lancet*, i, 212.
CLEMMESEN, J.—(1938) 'The Influence of X-radiation on the Development of Immunity to Heterologous Transplantation of Tumours.' London (Oxford University Press).
CONTAMIN, A.—(1910) *C. R. Acad. Sci.*, **150**, 128.
CRAMER, W.—(1932) *Sci. Rep. Cancer Res. Fd., Lond.*, **10**, 95.
DUNN, T. B.—(1945) Mammary Tumours in Mice, Symposium, *Amer. Ass. Adv. Sci.*, 13.
EHRLICH, P.—(1906) Arbeiten aus d. Koenigl, Inst. f. exp. Therapie, Frankfurt a M.
EISEN, M. J.—(1940) *Amer. J. Cancer*, **39**, 36.
GLÜCKSMANN, A.—(1948) *Brit. J. Radiol.*, **21**, 559.
GOLDFEDER, A.—(1941) *Radiology*, **37**, 705.—(1942) *Ibid.*, **39**, 426.—(1945*a*) *Ibid.*, **44**, 283.—(1945*b*) *Proc. Soc. exp. Biol., N.Y.*, **59**, 104.—(1947) *Radiology*, **49**, 724.—(1950*a*) *Ibid.*, **54**, 93.—(1950*b*) *Cancer Res.*, **10**, 89.—(1951) *Radiology*, **57**, 845.—(1952) *Cancer Res.*, **12**, 265.—(1953) *J. nat. Cancer Inst.*, Tissue Transp. Conference, **14**, 721.
*Idem* AND CAMERON, G.—(1948) *Cancer Res.*, **8**, 465.
HAALAND, M.—(1909) *Proc. Roy. Soc.*, B, **82**, 293.
KOLMER, J., AND TUFT, L.—(1941) 'Clinical Immunology, Biotherapy and Chemotherapy.' Monograph. London (Saunders Co.).
LAWRENCE, J. H., HORN, R., AND STRONG, L. C.—(1937) *Yale J. Biol. Med.*, **10**, 145.
LENZ, M.—(1942) *Radiology*, **38**, 686.
MOTTRAM, J. C., AND RUSS, S.—(1917) *Proc. Roy. Soc.*, B, **90**, 1.
STRONG, L. C., AND LITTLE, C. C.—(1920) *Proc. Soc. exp. Biol., N.Y.*, **18**, 43.
SUGIURA, K.—(1937) *Radiology*, **28**, 162.
WEDD, B. H., MORSON, A. C., AND RUSS, S.—(1914) *Arch. Middlesex Hosp.*, **33**, 71.